# Identification of New Genes Involved in Germline Predisposition to Early-Onset Gastric Cancer

**DOI:** 10.3390/ijms22031310

**Published:** 2021-01-28

**Authors:** Cristina Herrera-Pariente, Roser Capó-García, Marcos Díaz-Gay, Sabela Carballal, Jenifer Muñoz, Joan Llach, Ariadna Sánchez, Laia Bonjoch, Coral Arnau-Collell, Yasmin Soares de Lima, Mariano Golubicki, Gerhard Jung, Juan José Lozano, Antoni Castells, Francesc Balaguer, Luis Bujanda, Sergi Castellví-Bel, Leticia Moreira

**Affiliations:** 1 Centro de Investigación Biomédica en Red de Enfermedades Hepáticas y Digestivas (CIBEREHD), Gastroenterology Department, Institut d’Investigacions Biomèdiques August Pi i Sunyer (IDIBAPS), University of Barcelona, 08036 Barcelona, Spain; cristina.herrera@ciberehd.org (C.H.-P.); rosercg95@hotmail.com (R.C.-G.); mdiazgay@health.ucsd.edu (M.D.-G.); carballal@clinic.cat (S.C.); jenifer.munoz@ciberehd.org (J.M.); jllachr@clinic.cat (J.L.); asanchezg@clinic.cat (A.S.); bonjoch@clinic.cat (L.B.); arnau@clinic.cat (C.A.-C.); soaresdeli@clinic.cat (Y.S.d.L.); jung@clinic.cat (G.J.); castells@clinic.cat (A.C.); fprunes@clinic.cat (F.B.); 2 Department of Cellular and Molecular Medicine, University of California, San Diego, La Jolla, CA 92093, USA; 3 Oncology Section, Hospital of Gastroenterology “Dr. C. B. Udaondo”, C1264 Buenos Aires, Argentina; mariano.golubicki@gmail.com; 4 Molecular Biology Laboratory, Hospital of Gastroenterology “Dr. C. B. Udaondo”, C1264 Buenos Aires, Argentina; 5 Bioinformatics Platform, Centro de Investigación Biomédica en Red de Enfermedades Hepáticas y Digestivas (CIBEREHD), University of Barcelona, 08036 Barcelona, Spain; juanjo.lozano@ciberehd.org; 6 Centro de Investigación Biomédica en Red de Enfermedades Hepáticas y Digestivas (CIBEREHD), Gastroenterology Department, Biodonostia Health Research Institute, Basque Country University (UPV/EHU), 20014 San Sebastián, Spain; luis.bujanda@osakidetza.net

**Keywords:** gastric cancer, whole-exome sequencing, germline predisposition, early-onset, somatic profiling, next-generation sequencing

## Abstract

The genetic cause for several families with gastric cancer (GC) aggregation is unclear, with marked relevance in early-onset patients. We aimed to identify new candidate genes involved in GC germline predisposition. Whole-exome sequencing (WES) of germline samples was performed in 20 early-onset GC patients without previous germline mutation identified. WES was also performed in nine tumor samples to analyze the somatic profile using SigProfilerExtractor tool. Sequencing germline data were filtered to select those variants with plausible pathogenicity, rare frequency and previously involved in cancer. Then, a manual filtering was performed to prioritize genes according to current knowledge and function. These genetic variants were prevalidated with Integrative Genomics Viewer 2.8.2 (IGV). Subsequently, a further selection step was carried out according to function and information obtained from tumor samples. After IGV and selection step, 58 genetic variants in 52 different candidate genes were validated by Sanger sequencing. Among them, *APC*, *FAT4*, *CTNND1* and *TLR2* seem to be the most promising genes because of their role in hereditary cancer syndromes, tumor suppression, cell adhesion and *Helicobacter pylori* recognition, respectively. These encouraging results represent the open door to the identification of new genes involved in GC germline predisposition.

## 1. Introduction

With more than 1,000,000 new cases during 2018, gastric cancer (GC) is the fifth most common cancer worldwide, and ranks third in terms of cancer-related deaths [1]. The average age of diagnosis is near 60, about 6–7% of patients develop before the age of 50 and less than 2% before 40 [2]. Lauren’s classification is one of the most used, subdividing GC in two histological types, intestinal and diffuse. The first one is characterized by tubular and glandular structures and is more common in older patients. In the second one, tumor cells lack cell-to-cell interaction, being more common in young patients [3]. Both genetic and environmental factors are involved in GC predisposition. Most GC cases are sporadic, caused by *Helicobacter pylori* infection, age, tobacco and alcohol consumption and dietary habits (such as a diet rich in fried food, red meat and low consumption of fresh fruits and vegetables), among others [4,5]. However, at least 10% of total GC cases display familial aggregation, and a genetic cause is present in up to 5% of all cases [6].

The most common inherited form is hereditary diffuse gastric cancer (HDGC) syndrome, which is characterized by early-onset, multigenerational diffuse GC and lobular breast cancer. It is mainly caused by *CDH1* germline mutations (encoding E-cadherin protein), explaining at least 20% of the cases [7], and less frequently by *CTNNA1* [8]. Other conditions involved directly in GC development are gastric adenocarcinoma and proximal polyposis of the stomach, caused by *APC* promoter 1B mutations [9], and familial intestinal gastric cancer syndrome (FGC), although no inherited cause has been already identified. In addition, GC is also associated with other hereditary cancer syndromes such as Lynch, Li-Fraumeni, familial adenomatous polyposis, Peutz–Jeghers, hereditary breast and ovarian cancer and juvenile polyposis, with mutations in mismatch repair genes, *TP53*, *APC*, *STK11*, *BRCA1*/*2* and *SMAD4*/*BMPR1A*, respectively [6].

In spite of that, the germline cause for several families with GC aggregation remains unclear. Identifying those genes is especially important in early-onset GC patients (EOGC), defined by a diagnosis at 50 years old or earlier. Only 10% of them have a positive family history that is explained by the hereditary cancer syndromes previously mentioned [10]. The remaining 90% of cases do not show a family history, hampering early diagnosis and decision making. Additionally, EOGC differs from conventional GC not only clinico-pathologically but also at the molecular level, having for example a different somatic mutation frequency profile in particular genes when compared to conventional GC [11]. Clinico-pathologically, EOGC are usually diagnosed at an advanced stage, associating a high mortality, and unlike GC in advanced ages, there is a predominance of diffuse histology, proximal location, without a clear gender predominance, infrequent association with intestinal metaplasia or somatic loss of RUNX3 and commonly associated with gains on chromosomes 17q, 19q and 20q [10]. Furthermore, these patients have been less exposed to those environmental factors involved in GC development. So, focusing on this subgroup of patients is a useful approach in order to discover their germline background [10]. Indeed, *CDH1* germline mutations are an uncommon event in EOGC patients [12], supporting the idea that other genes may be involved in GC predisposition in this subgroup of patients.

During last years, with the emergence of next-generation sequencing technologies, the identification of genetic variation has been facilitated. Specifically, whole-exome sequencing (WES) is a cost-effective approach that allows sequencing the coding region of the genome. The vast majority of studies have been focused on somatic analysis in order to identify the most recurrent mutated genes and those who are involved in GC carcinogenesis [13]. However, this technique has also permitted to achieve promising but heterogeneous results in the identification of new genes involved in GC germline predisposition, although molecular background of hereditary GC is not fully understood [7,14,15,16,17,18].

Bearing in mind that germline background is higher in EOGC patients than conventional GC patients, the aim of the present study was to identify new candidate genes involved in germline predisposition to GC, using WES of germline samples of EOGC patients, without *CDH1* germline mutations.

## 2. Results

### 2.1. Clinico-Pathological Features of the Cohort

Twenty patients with GC diagnosed before aged 51 were included. Clinico-pathological features of patients included in the study are summarized in Table 1.

The median age at diagnosis was 41.5 years old (interquartile range, IQR 34–46), with a predominance of women with 13 (65%) cases. None of the patients had a history of another tumor. A total of 5 (25%) patients presented familiar aggregation of GC (first, second or third grade relative) and none met criteria for FGC. A total of 10 (50%) patients had family history of other cancers (highlighting 3 relatives with pancreatic cancer and 2 with lung cancer).

The predominant tumor histology was diffuse, observed in 16 (80%) of the cases. Regarding tumor location, the most common site was the body in 13 (65%). An advanced stage (III/IV) at diagnosis was present in 7 (35%) cases.

### 2.2. Mutational Profile Analysis

**Figure 1:**
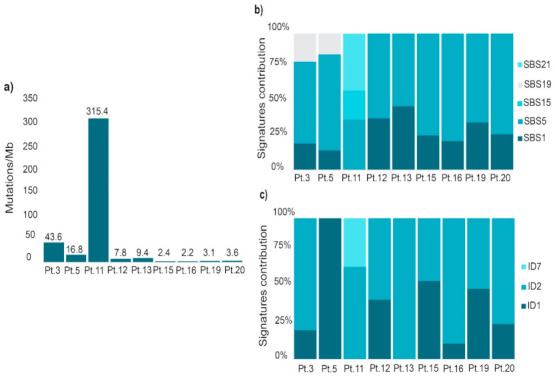
Somatic mutational profile analysis performed with SigProfilerExtractor in nine GC tumor samples. (**a**) tumor mutational burden (number of mutations per megabase). (**b**) single base substitution (SBS) signatures contribution in each sample. (**c**) indel signatures contribution in each sample. Pt, patient; Mb, megabase; ID, small insertions and deletions.

Somatic WES was performed in nine available tumor samples (one per patient) and mutational profile was evaluated using SigProfilerExtractor. Results are shown Figure 1. Regarding tumor mutational burden (TMB) (number of mutations per megabase), patient 11 sample, with 315.4 mutations per megabase, can be classified as an ultra-hypermutated tumor (>100 mutations/megabase) [19]. Among hypermutated samples (>10 mutations/megabase), patients 3 and 5 samples, with 43.6 and 16.8 mutations per megabase, respectively, can be highlighted (Figure 1a).

Regarding Catalogue of Somatic Mutations in Cancer (COSMIC) signatures contribution, all samples are characterized by single base substitution (SBS) 1 and SBS5, which are clock-like signatures, correlated with the age of the individual (Figure 1b). However, samples of patients 3, 5 and 11 displayed other signatures. Pt.3 and Pt.5 samples showed SBS19, with unknown etiology and never identified in GC samples in the ICGC/TCGA Pan-Cancer Analysis of the Whole Genome (PCAWG) project. Interestingly, Pt.11 sample is also characterized by SBS15 and SBS21. Both are involved in defective DNA mismatch repair, and SBS15 has been previously identified in 12% of GC samples of the ICGC/TCGA PCAWG project. Focusing on small insertions and deletions (ID) signatures (Figure 1c), all samples showed ID1, ID2 or both. These signatures are correlated with the age of cancer diagnosis in non-hypermutated samples. However, they tend to be elevated in samples with defective DNA mismatch repair. Additionally, Pt.11 sample also showed ID7, involved in defective DNA mismatch repair.

### 2.3. Germline Analysis

**Figure 2:**
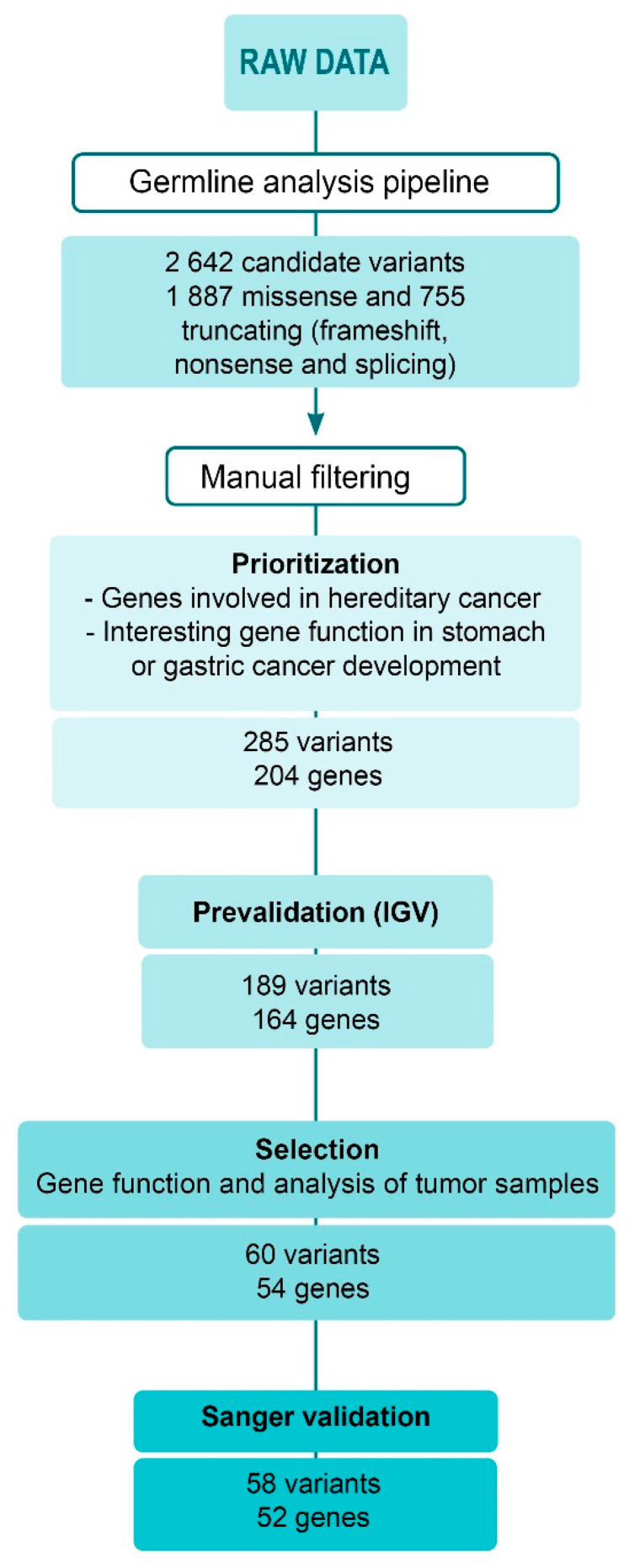
Scheme of the germline data analysis after whole-exome sequencing. Germline analysis pipeline characteristics can be found in Figure S1 in Supplementary Materials.

After germline pipeline analysis, 2642 different candidate variants remained, some of them shared by several patients. Among them, 1887 were missense variants, while the remaining 755 included nonsense, frameshift and splice variants. In order to reduce the amount of candidate variants to validate in further steps, a manual filtering was performed (Figure 2).

**Figure 3:**
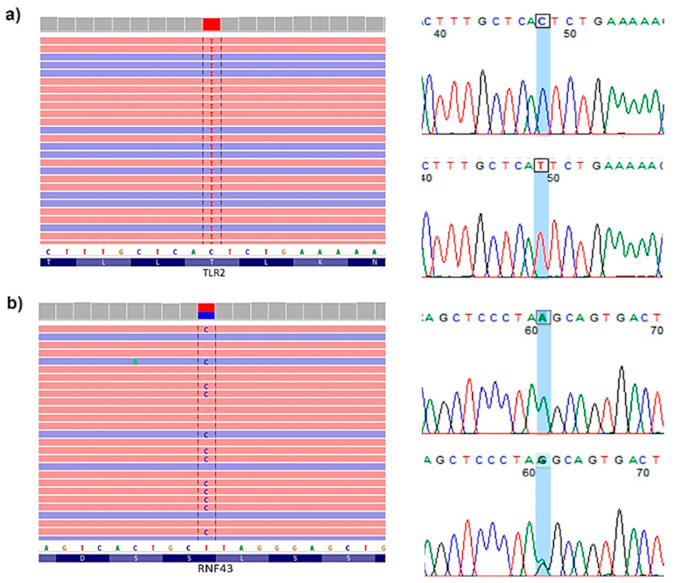
Example of prevalidation and Sanger sequencing of recessive and dominant candidate variants. (**a**) prevalidation by Integrative Genomics Viewer 2.8.2 (IGV) and validation by Sanger sequencing of homozygous *TLR2* variant (c.1232C>T) of control (up) and patient from family 4 (down). (**b**) prevalidation by IGV and validation by Sanger sequencing of heterozygous *RNF43* variant (c.1504A>G) of control (up) and patient from family 4 (down).

After a prioritization step, 285 candidate variants located on 204 different genes fulfilled the established criteria in both recessive and dominant inheritance analysis. In the first one, 72 candidate variants remained, while in dominant analysis 213 variants were prioritized, located on 29 and 177 genes, respectively. Subsequently, a prevalidation step using Integrative Genomics Viewer 2.8.2 (IGV) was performed. In this step, 96 variants were discarded because they were doubtful variants located in non-coding or repetitive regions (like T-tracks) or because few reads identified them. Among the remaining 189 prevalidated variants (8 recessive and 181 dominant), 60 were selected considering gene function and somatic mutational profile analysis. These variants were located on 54 different candidate genes. Two candidate variants belonged to a recessive pattern of inheritance and the remaining 58 were inherited dominantly. Several genes had different candidate variants, and, specifically *TLR2*, was shared by both recessive and dominant analysis, with different variants in different patients. After primer design, Sanger sequencing was performed in the selected 60 variants. Afterwards, 58 were positively validated by Sanger sequencing (Supplementary Table S1). Examples of IGV prevalidation and Sanger sequencing results for recessive and dominant variants are shown in Figure 3a,b, respectively.

**Figure 4:**
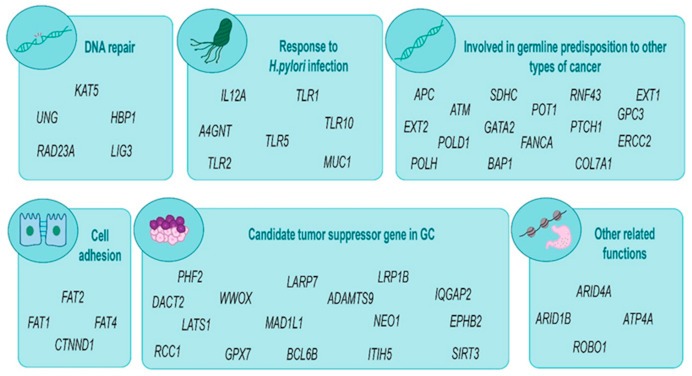
Gastric cancer predisposition candidate genes classify by function or involvement in hereditary cancer.

The 58 validated genetic variants were located on 52 genes. These candidate genes can be classified according to gene function (such as cell adhesion, response to *H. pylori* infection, DNA repair or tumor suppression) or previous association with hereditary cancer (Figure 4).

## 3. Discussion

WES of germline and available tumor samples was performed in 20 unrelated patients with GC before the age of 51 with the aim to identify novel GC susceptibility genes. Finally, 58 candidate variants located on 52 genes remained.

It is well known that DNA repair is an important function in cancer. In the present study, somatic mutational profile analysis identified that patient 11 displayed defective DNA mismatch repair signatures and had a high TMB (ultra-hypermutated sample). This suggests that variation in DNA repair genes could be involved in GC development in this individual, more strongly than in the other patients. The validated candidate variants in this patient were located on *ERCC2*, *GATA2* and *HBP1* genes, involved in excision repair, transcriptional regulation and cell cycle inhibition, respectively. Additionally, *HBP1* downregulation has been related with delay in DNA repair [20]. Although *ERCC2* and *HBP1* are not related to mismatch repair, they are involved in DNA repair, so they could be interesting candidate genes to bear in mind for this patient in further steps. Interestingly, a deficiency in mismatch repair system was observed in the tumor of this patient and Lynch syndrome was discarded. This patient was a 48-year-old man with intestinal GC without previous cancer family history; the tumor showed loss of protein expression of MLH1 and PMS2 and the germline genetic analysis rule out a mutation in Lynch syndrome associated genes.

Regarding the two hypermutated samples, Pt.3 and Pt.5, both showed a mutational signature with unknown etiology and without previous association with GC samples. Interestingly, in Pt.3 a variant was identified in *RAD23A*, a gene involved in nucleotide excision repair function. The remaining candidate variants identified in these patients are located on *EXT1*, *SDHC* and *TLR10* for Pt.3 and on *APC*, *TLR5* and *WWOX* in Pt.5. 

As it is well known, *POLH* and *POLD1* genes are involved in DNA repair. In the present study, variants in these genes have been identified in Pt.9 and Pt.15, respectively, although no correlation with TMB or DNA mismatch repair signatures have been identified in any of them. 

Cell adhesion is another important function in GC development, since *CDH1*, the most important germline predisposing gene, is involved in intercellular adhesion. Indeed, it has been found that cell adhesion is the most significantly enriched biological process among the mutated genes in GC tumor exomes. Among related genes, *FAT4* is one of the most recurrently mutated in up to 75% of GC samples [13,21,22]. Importantly, it is known that 10% of somatically mutated genes can confer susceptibility to cancer when mutated at germline level [23], so *FAT4* could be an interesting candidate gene. A missense variant in *FAT4* was validated in the present study. Variants in *FAT1* and *FAT2* were also identified, although *FAT4* has stronger evidence supporting a role in gastric carcinogenesis. *FAT4* is a member of the cadherin superfamily and it is involved in planar cell polarity, the Hippo signaling pathway, the canonical Wnt signaling cascade, and the expression of YAP1 [24]. Mutations in this gene have been identified in several types of cancer [25]. It has been also associated with poor prognosis in GC patients [26] and in vitro and in vivo experiments found that *FAT4* knockdown increases tumor growth and metastasis, mediated by the Wnt/β-catenin signaling pathway [22]. This fact supports the idea that it is not only involved in cell adhesion, but also in tumor suppression in GC. Interestingly, germline mutations in this gene have been found in patients with Van Maldergem syndrome or Hennekam syndrome, but in both cases with a recessive inherited pattern [27]. Altogether, potentially pathogenic variants in this gene are interesting candidates to be involved in germline GC predisposition.

*CTNND1*, also known as *p120*, is an E-cadherin regulator that belongs to the catenin family. It is known that its expression is altered in several human cancer types [28,29]. Although, some studies claimed that catenin genes do not play a key role in HDGC [30], other family member, *CTNNA1*, has been involved in GC germline predisposition [8]. The CTNND1 protein is able to bind to the E-cadherin cytoplasmatic domain preventing the entry of E-cadherin into degradative pathways. It is crucial to maintain E-cadherin levels in plasma membrane to mediate cell to cell interactions [31]. It has been reported that mutations in *CDH1* that affect CTNND1 binding domain make E-cadherin degradation more likely [32], so their binding is important for cell adhesion. Another interesting aspect is that in vitro studies identified that *CTNND1* knockdown promoted cell proliferation and invasion in GC cell lines [33]. However, other study suggested that *CTNND1* knockdown reduced cell migration and invasion and no influence on cell proliferation was detected [34]. So, more studies will be needed in order to clarify *CTNDD1* role in GC. 

Another interesting function to bear in mind in GC predisposition is response to *H. pylori* infection and its recognition, since it is an important risk factor. The Toll-like receptor (TLR) signaling pathway is crucial in pathogen recognition and activation of innate immunity. In the present study, variants in *TLR1*, *TLR2*, *TLR5* and *TLR10* were validated. However, there is a strong evidence supporting a crucial role for *TLR2* and increased levels have been found during *H. pylori* infection [35]. *TLR2* recognizes *H. pylori* lipopolysaccharide, causing chemokine secretion by gastric epithelial cells, and finally, activating NF-κB [36]. However, its role is controversial. Yokota et al., described that *TLR2* activation leads to proliferation of gastric epithelial cells and strong inflammatory reaction [37]. Additionally, a *TLR2*-upregulated gene expression signature that correlates with impaired GC survival has been described [38]. However, other studies claimed that *TLR2* deficiency may be harmful and implicated in gastric carcinogenesis, because weaken immune response would take place and infection would increase [35,39]. Furthermore, polymorphisms in this gene have been associated with an increased risk of GC, but sometimes in an ethnic-specific manner [40,41]. Discovering the role of *TLR2* in GC predisposition is still necessary. In this study, two variants have been validated.

A variant in *MUC1* was also validated. Its main function is forming mucous barriers on epithelial surfaces and different variants have been associated with diffuse GC [42]. Some studies suggest that can confer a moderate-low risk to GC, although some evidences claimed that it may act as an oncogene [6].

Tumor suppression has a key role in cancer development, and loss of function of tumor suppressor genes is involved in germline predisposition to cancer. In the present work, several genes involved in this function were identified. For example, three different variants in *WWOX* were validated in this study. *WWOX* is frequently altered in cancer and it is considered a tumor suppressor gene [43]. In GC, focal deletions are commonly found [44]. Additionally, a variant in *BCL6B* was validated. This gene has been involved in tumor suppression in GC and epigenetic inactivation via its own promoter hypermethylation is involved in GC development [45]. Furthermore, its downregulation in combination with a severe inflammatory response is correlated with poor survival in GC patients [46]. Finally, *LATS1* has been associated with essential life functions such cell proliferation, apoptosis and migration. In vivo experiments showed that in GC tissues its overexpression suppressed cell growth and tumorigenicity [47]. All these genes seem to be interesting candidates for GC predisposition according to their function, although future studies are needed in order to stablish their role in germline predisposition. 

Regarding those genes involved in germline predisposition to other cancers, *ATM*, *APC*, *POLD1* or *SDHC* could be promising candidate genes for germline GC predisposition. They are involved in germline predisposition to breast cancer, familial adenomatous polyposis, colorectal cancer and gastrointestinal stromal tumor, respectively. Although, patients with candidate variants in these genes did not present family history related to these syndromes, these variants could be explained by de novo mutations, incomplete penetrance or “non-informative” families due to small family size or lack of accurate information. So, it does not restrict the idea that these genes could be involved in germline predisposition to GC. Among them, *APC* is one the strongest candidate because it is involved in familial adenomatous polyposis, a syndrome that can also cause GC. In addition, *APC* is involved in Wnt signaling pathway regulation and dysregulation of this pathway has been involved in development of almost 30% of GC cases [48]. 

Regarding other studies, WES has been a common strategy in order to identify new genes involved in germline predisposition. A study with GC patients without genetic testing or uninformative identified variants in genes involved in DNA damage response pathway, such as *ATM*, *ATR*, *BRIP1*, *FANCC* or *TP53*, among others [14]. Three different studies identified that patients with HDGC syndrome, without *CDH1* germline mutations, presented mutations in genes such as *CTNNA1*, *BRCA2*, *STK11*, *PALB2*, *RAD51C*, *BRCA1*, *RECQL5*, *MSH2*, *ATR* and *NBN* [7,15,16]. Despite advances in this field, few studies have focused on young patients. Vogelaar et al. did not find any clear novel GC predisposing gene in EOGC patients and some of their relatives, probably because of the heterogeneity of the cohort [17]. Other study, using targeted-sequencing, concluded that genetic variants in genes such as *CTNNA1* or *MYD88* are infrequent in EOGC patients and discarded *MAP3K6* as a candidate gene in GC predisposition [18]. 

On the other hand, in order to reduce the number of potential candidate genes different strategies can be followed. The present study prioritized genes that could be involved in GC development or hereditary cancer. Similar strategy was performed by Vogelaar and colleagues [17] selecting those genes involved in cancer predisposition, GC development, immunodeficiency predisposition and high expression in stomach. Other study selected pathogenic or likely pathogenic variants [14]. 

The main strength of our study is that it is focused on young GC patients. These patients have been less exposed to environmental factors at the age of diagnosis, suggesting that their genetic background may have a stronger role in cancer development. So, focusing on young patients is a useful strategy in order to identify their germline background. In addition, the present study has evaluated not only germline but also tumor samples in almost half of the cohort, what has not been previously performed by other groups, to our knowledge. 

The identification of germline variants associated to gastric cancer will help not only identify GC-high risk populations in order to establish preventive strategies and early diagnosis, but also it could help to tailored treatment strategies. During the last years, there is increasing evidence of the benefit of personalized medicine based on the presence of germline mutations, as it is already demonstrated in other tumors (such as breast, ovary and pancreas) in association with *BRCA* [49,50,51]. For example, focusing on GC, it would be interesting to analyze if specific germline mutations have influence in the response to capecitabine as adjuvant therapy in advanced GC patients [52].

However, the present study has some limitations. First, information regarding *H. pylori* infection status was not available. This hampered to establish a relationship between those candidate genes involved in response to *H. pylori* infection and GC development in these patients. Additionally, this is an exploratory study, and our results are preliminary. So, replication in a larger independent cohort and functional studies will be needed to further confirm the role of these candidate genes in GC germline predisposition.

## 4. Materials and Methods

### 4.1. Patients

Twenty unrelated patients (one per family) who developed GC before the age of 51 were recruited from Hospital Clínic in Barcelona and Hospital Donostia in San Sebastián. *CDH1* germline mutations were discarded in all patients. Copy number alterations such as deletions or duplications in *CDH1* were also discarded using CoNVaDING tool [53]. Additionally, a commercial multigene panel (Trusight Cancer v1, Illumina Inc., San Diego, CA, USA) was used in those patients that fulfill clinical criteria for other hereditary cancer syndromes in order to discard them. Germline DNA samples were obtained from peripheral blood, whereas somatic DNA samples were available in 9 patients, from paraffin-embedded tumor. QIAamp DNA Blood Kit or QIAamp Tissue Kit (Qiagen, Redwood City, CA, USA), were used, respectively, according to the manufacturer’s instructions. Personal and family history data was also obtained.

### 4.2. Whole-Exome Sequencing and Bioinformatic Analysis

WES was performed with germline DNA and, when available, somatic DNA, using the HiSeq 2000 platform (Illumina, San Diego, CA, USA) and the SureSelect Human All Exon V5 kit (Agilent Technologies, Santa Clara, CA, USA) for exon enrichment. Mass parallel sequencing was done with a reading length protocol of 2 × 100 base pairs and a minimum coverage of 70×. Consecutively, the alignment on the human genome (hg19/GRCh37 for germline samples and hg38/GRCh38 for somatic samples) was performed using Genome Multitool.

For variant calling, HaplotypeCaller tool from the Genome Analysis Toolkit (GATK) were used for germline samples. For somatic samples, MuTect, Strelka, VarScan and MuSe tools were used. Variant annotation took into account data available in dbSNP (http://www.ncbi.nlm.nih.gov/SNP/), the 1000 Genomes Project (http://www.1000genomes.org) and the Exome Variant Server (http://evs.gs.washington.edu). Data regarding functional consequences of the variant (stop codon, missense, synonymous, splicing, frameshift or structural interaction) and genome position were annotated through the SnpEff tool (http://snpeff.sourceforge.net). In addition, for missense variants a group of different pathogenicity predicting tools (PhyloP (http://compgen.bscb.cornell.edu/phast/help-pages/phyloP.txt), Combined Annotation Dependent Depletion (CADD; https://cadd.gs.washington.edu/), likelihood ratio test (LRT), MutationTaster (http://www.mutationtaster.org), PolyPhen2 (http://genetics.bwh.harvard.edu/pph2) and Sorting Intolerant From Tolerant (SIFT; http://sift.bii.a-star.edu.sg)) were used. For germline samples, an in-house R language pipeline [54] was used to annotate also biological functions and pathways of the genes containing each variant with terms and previous bibliography according to NCBI Gene (http://www.ncbi.nlm.nih.gov/gene), Gene Ontology (http://geneontology.org/), KEGG (http://www.genome.jp/kegg/), and Reactome (http://www.reactome.org/PathwayBrowser/). Using these databases, a list of several terms was created (Supplementary Table S2) and used to select variants. This in-house pipeline also prioritized regarding functional effect (missense and nonsense, splicing or indels genetic variants) and selected them according to its gene function on the public databases previously mentioned, sequence quality and population frequency in order to discard polymorphisms. The workflow is displayed in Supplementary Figure S1.

### 4.3. Mutational Profile Analysis of Tumoral Samples

In order to evaluate the mutational profile of tumor samples, SigProfilerExtractor (https://github.com/AlexandrovLab/SigProfilerExtractor) [55] was used in the nine available samples. This tool allows the characterization of the profile and TMB, as well as the quantification of the mutational signature contribution reported in the COSMIC database (https://cancer.sanger.ac.uk/cosmic/signatures/index.tt) (v3.1 June 2020). This tool also permits to associate patterns of mutations with cellular processes or external agents that are causing them, helping us to select the most suitable candidate genes that could be involved in germline predisposition. Somatic variants identified by two out of four variant callers were used for single nucleotide variants and two out of three, for indels analysis.

### 4.4. Variant Prioritization

After pipeline filtering of germline data, manual filtering was performed to complete the prioritization of the variants. In the first step of the manual filtering, those variants located on genes previously involved in germline predisposition to any type of cancer were selected [23].

In the second step, the criteria of gene prioritization were based mainly on the current knowledge and gene function. To that end, public databases such as NCBI Gene, Pubmed and Online Mendelian Inheritance in Man (OMIM) were consulted in order to know its function, latest available information and if they were involved in human genetic disorders, respectively. Both recessive and dominant patterns of inheritance were considered. Regarding recessive inheritance, those genes related with cancer and pathogen recognition were selected, because of the important role on *H. pylori* in GC development. In relation to dominant inheritance, genes related with stomach functioning or gastric cancer development were filtered. Among all of them, functions such as tumor suppression, DNA repair, apoptosis, pathogen recognition, innate immunity activation, gut epithelial protection or cell adhesion-related genes were prioritized. Other functions such as alcohol metabolism or gastric acid secretion were also considered because they are GC risk factors. Additionally, variants that appeared in more than 25% of patients were not considered.

### 4.5. Variant Prevalidation, Selection and Final Validation

Prioritized variants were prevalidated using the IGV (http://software.broadinstitute.org/software/igv/) [56] by manual inspection of the WES data. It is a high-performance visualization tool for next-generation sequencing data that permits to discard sequencing errors and/or strand bias. Variants in non-coding or repetitive regions or with few reads identifying it were discarded. Subsequently, selection of most interesting and positively prevalidated genetic variants was done. In this step, information obtained from the analysis of tumor samples and gene function was considered.

Final candidate genetic variants were validated by Sanger sequencing. Primers were designed using Primer3 v.0.4.0. (http://bioinfo.ut.ee/primer3-0.4.0/). Primer sequences are listed in Supplementary Table S3. After PCR amplification, Sanger sequencing was carried out by Eurofins Genomics and resulting sequences were visualized using FinchTV (https://finchtv.software.informer.com/1.4/).

## 5. Conclusions

Taken together, we could conclude that the most promising candidate genes in GC germline predisposition are involved in functions such cell adhesion, *H. pylori* recognition, tumor suppression and germline predisposition to hereditary cancer. Those genes could be *FAT4*, *CTNND1*, *TLR2* and *APC*. Although replication in an external cohort and functional experiments are needed, these encouraging results represent the open door to the identification of new genes involved in GC germline predisposition.

## Figures and Tables

**Table 1:** Clinico-pathological features of the included patients (*n* = 20).

**Age at cancer diagnosis**; median (IQR)	41.5 (34.2–46.5)
**Gender** (women); number (%)	13 (65)
**Personal history of other neoplasm**, number (%)	0
**Family history of GC**	
(FDR, SDR, TDR), number (%)	5 (25)
Age at cancer diagnosis; median (range)	61 (46–70)
Familial gastric cancer criteria, number (%)	0
**Family history of other tumors** (FDR, SDR, TDR), number (%)	10 (50)
Pancreas	3 (15)
Lung	2 (10)
Brain	1 (5)
Bone	1 (5)
Leukemia	1 (5)
Liver	1 (5)
Endometrium	1 (5)
Tumor location, number (%)	
Cardias	1 (5)
Fundus	3 (15)
Body	13 (65)
Antrum	3 (15)
Tumor stage, number (%)	
I/II	13 (65)
III/IV	7 (35)
GC histology, number (%)	
Diffuse	16 (80)
Intestinal	4 (20)
Tumor differentiation grade, number (%) *	
Intestinal histology (*n* = 4):	
High-grade (poorly differentiated)	1 (25)
Low grade (well-moderately differentiated)	3 (75)

IQR, interquartile range; GC, gastric cancer; FDR, first-degree relative; SDR, second-degree relatives; TDR, third-degree relatives. * According to the WHO classification (2019), the degree of differentiation only applies to the tubulo-papillary phenotype of GC (intestinal type).

## Data Availability

Not applicable.

## Supplementary Materials

Supplementary materials can be found at https://www.mdpi.com/1422-0067/22/3/1310/s1. Table S1: Final selected variants including functional information and population frequency. Genes marked with an asterisk (*) correspond to those previously involved in germline predisposition to other cancers. Table S2: Key terms used to prioritize genetic variants with functional annotations and/or bibliography related to cancer. Functional annotations listed corresponded to those selected from Gene Ontology, KEGG and REACTOME, whereas the Bibliography category referred to terms from NCBI gene summary and Gene Reference Into Function (GeneRIF). Noteworthy, other annotations containing listed key terms were also selected (e.g., “breast cancer” since it contains “cancer”). Table S3: Primer sequence for Sanger sequencing validation. Figure S1: Workflow of pipeline germline analysis.
