# Supplementary material for: Identification of New Genes Involved in Germline Predisposition to Early-Onset Gastric Cancer
Source: Int J Mol Sci. 2021 Jan 28;22(3):1310. doi: 10.3390/ijms22031310 (PMC7866206; doi:10.3390/ijms22031310)
Supplement: Supplementary file 1 [file ijms-22-01310-s001.zip › supp/Table S1-checked_27012021.DOCX]

**Table S1.** Final selected variants including functional information and population frequency. Genes marked with an asterisk (*) correspond to those previously involved in germline predisposition to other cancers.

| **Gene** | **Het/Hom** | **Variant** | **Patient**  **ID (GC Histology)** | **Classification** | **Function** | **gnomAD** | **ClinVar** |
| --- | --- | --- | --- | --- | --- | --- | --- |
| *ADAMTS9* | Het | c.4259T>C (p.Leu1420Ser) | 4 (D) | Missense | Cleavage of proteoglycans, control of organ shape and angiogenesis. Functional TS in GC | 2/251248 | - |
| *APC** | Het | c.7262C>T (p.Ser2421Leu) | 5 (D) | Missense | TS that acts as an antagonist of Wnt signaling pathway. Involved in familial adenomatous polyposis | 3/250222 | Uncertain significance |
|  |  | c.6958C>T (p.Pro2320Ser) | 15 (D) | Missense |  | 1/250878 | Uncertain significance |
| *ARID1B* | Het | c.3760A>G (p.Met1254Val) | 14 (D) | Missense | Component of SWI/SNF chromatin remodelling complex. Similar to ARID1A, commonly mutated in GC | 19/282876 | - |
| *ARID4A* | Het | c.449T>G (p.Val150Gly) | 9 (D) | Missense + splice region | Transcriptional repression activity. TS in a variety of malignancies. Downregulated in GC | 1/250566 | - |
| *ATM** | Het | c.68G>A (p.Arg23Gln) | 17 (D) | Missense | Cell cycle checkpoint kinase. Involved in breast cancer predisposition. | - | Uncertain significance |
| *ATP4A* | Het | c.2002C>T (p.Arg668Cys) | 16 (D) | Missense | Proton pump involved in gastric acid secretion | 56/281398 | - |
| *A4GNT* | Het | c.719A>G (p.Asp240Gly) | 2 (D) | Missense | O-glycan transferase that acts on mucus suppressing *H. pylori* growth. | - | - |
| *BAP1* | Het | c.1268C>A (p.Thr423Lys) | 17 (D) | Missense | Deubiquitinating enzyme. Germline mutations associated with tumor predisposition syndrome. Decreased expression suggests bad prognosis for GC patients | 147/282716 | Benign/Likely benign |
| *BCL6B* | Het | c.173C>T (p.Ala58Val) | 2 (D) | Missense | Transcriptional repressor. Novel functional TS in GC | - | - |
| *COL7A1** | Het | c.7334C>T  (p.Pro2445Leu) | 1 (D) | Missense | Anchoring fibril. Involved in cell squamous cancer predisposition | 4/223706 | - |
| *CTNND1* | Het | c.28_29delinsCT (p.Ala10Leu) | 9 (D) | Missense | Cell adhesion and signal transduction. Regulator of endocytic process of *CDH1* | 102/235764  102/239110 | - |
| *DACT2* | Het | c.1784C>A (p.Ala595Glu) | 6 (D) | Missense | Negative regulation in Wnt/β-catenin and Nodal/TGFβ signalling pathway. TS in colon cancer. Methylated in GC | 10/183992 | - |
| *EPHB2* | Het | c.847G>C (p.Asp283His) | 17 (D) | Missense | Involved in motility division and differentiation. Emerging TS gene in CRC. Inactivation in the stomach may contribute to tumor formation | 70/282810 | - |
| *ERCC2** | Het | c.1775G>A (p.Arg592His) | 11 (I) | Missense | Excision repair. Involved in melanoma predisposition | 104/282486 | Not provided |
| *EXT1** | Het | c.770C>T (p.Pro46Leu) | 3 (D) | Missense | Heparan sulfate biosynthesis. Involved in chondrosarcoma predisposition | - | - |
| *EXT2** | Het | c.965G>A (p.Arg355His) | 7 (I) | Missense | Heparan sulfate biosynthesis. Involved in chondrosarcoma predisposition | 179/282846 | - |
| *FANCA** | Het | c.98C>G (p.Ser33Trp) | 4 (D) | Missense | DNA replication and damage response. Involved in Fanconi anemia pathway and in leukemia predisposition | - | - |
| *FAT1* | Het | c.10195G>A (p.Asp3399Asn) | 14 (D) | Missense | Adhesion molecule and/or signaling receptor. Ortholog of *D. melanogaster* fat gene TS | 7/246924 | - |
| *FAT2* | Het | c.3311A>G (p.Glu4332Gly) | 18 (D) | Missense | Adhesion molecule and cell proliferation control. Ortholog of *D. melanogaster* fat gene TS | 9/250062 | - |
| *FAT4* | Het | c.2186C>A (p.Ser729Tyr) | 1 (D) | Missense | Planar cell polarity, cell adhesion and TS in GC | 1/249424 | - |
| *GATA2** | Het | c.445G>A (p.Gly149Arg) | 11 (I) | Missense | Transcriptional regulator. Involved in leukemia predisposition | 24/219956 | Conflicting interpretations of pathogenicity |
| *GPC3** | Het | c.167C>T (p.Pro56Leu) | 17 (D) | Missense | Control of cell division and growth regulation. Involved in hepatoblastoma predisposition | - | - |
| *GPX7* | Het | c.137C>T (p.Ser46Leu) | 4 (D) | Missense + splice region | Intracellular sensor and redox detector. Hypermethylated in GC. Potential TS in esophageal adenocarcinoma | - | - |
| *HBP1* | Het | c.1167A>C (p.Gln389His) | 11 (I) | Missense | Transcription factor. *HBP1* repression delays DNA damage repair and causes cell death | 35/251370 | - |
| *IL12A* | Het | c.556C>A (p.Gln186Lys) | 7 (I) | Missense | Pro-inflammatory cytokine. Its lack results in a reduction in host resistance to infections | - | - |
| *IQGAP2* | Het | c.4582G>A (p.Val1528Ile) | 9 (D) | Missense | TS and regulating innate antiviral responses role. Aberrant promoter methylation in gastric cancer cells | - | - |
| *ITIH5* | Het | c.1400C>T (p.Ala467Val) | 4 (D) | Missense | Extracellular matrix stabilization and prevention of tumor metastasis. Decreased expression is associated with poor prognosis in primary GC | - | - |
| *KAT5* | Het | c.478G>C (p.Gly160Arg) | 12 (D) | Missense | DNA repair, apoptosis and signal transduction. Its downregulation is important for the malignant pathway of gastric carcinogenesis | 36/282218 | - |
| *LARP7* | Het | c.778A>G (p.Arg260Gly) | 9 (D) | Missense | Transcriptional regulator.  Potential TS gene in GC | - | - |
| *LATS1* | Het | c.286C>T (p.Arg96Trp) | 16 (D) | Missense | Cellular homeostasis. TS role. Negative expression associated with poor overall survival | 995/282700 | - |
| *LIG3* | Het | c.86G>A (p.Trp29*) | 1 (D) | Stop gained | Excision repair | 3/251438 | - |
| *LRP1B* | Het | c.7484G>A (p.Arg2495Gln) | 8 (D) | Missense | Role in normal cell function and development. TS gene. Hypermethylation in GC | 12/281420 | - |
| *MAD1L1* | Het | c.787C>T (p.Arg263Trp) | 4 (D) | Missense | Role in cell cycle control and TS. Candidate tumor suppressor gene in GC | 62/279966 | - |
| *MUC1* | Het | c.758C>T (p.Ser253Phe) | 6 (D) | Missense | Protective mucous barriers formation on epithelial surfaces | 1/31390 | - |
| *NEO1* | Het | c.4090G>T (p.Val1364Leu) | 6 (D) | Missense | Cell growth, proliferation and cell-cell adhesion | 2/251324 | - |
| *PHF2* | Het | c.2188A>G (p.Ser730Gly) | 7 (I) | Missense + splice region | Lysine demethylase. TS gene in association with p53. Frameshift mutations in GC with MSI-H | 15/279976 | - |
| *POLD1** | Het | c.931C>T (p.Arg311Cys) | 15 (D) | Missense | Critical role in DNA replication and repair. Involved in CRC predisposition | 4/274548 | Uncertain significance |
| *POLH** | Het | c.698A>G (p.Asn233Ser) | 9 (D) | Missense | Polymerase. Involved in cell squamous cell predisposition | 217/282890 | Likely benign |
| *PTCH1** | Het | c.3924A>T (p.Arg1308Ser) | 8 (D) | Missense | Component of the hedgehog signaling pathway Involved in basal cell carcinoma predisposition | 2/246494 | Uncertain significance |
| *POT1* | Het | c.143C>T (p.Thr48Ile) | 19 (D) | Missense | Telomere maintenance. Susceptibility to melanoma | - | - |
| *RAD23A* | Het | c.434G>A (p.Gly145Asp) | 3 (D) | Missense | Nucleotide excision repair | 194/265936 | - |
| *RCC1* | Het | c.206T>C (p.Val100Ala) | 7 (I) | Missense | Critical cell cycle regulator. Hypermethylated in GC. May play a TS role in GC | 13/238056 | - |
|  |  | c.508C>T (p.Arg170Cys) | 10 (D) | Missense |  | 11/279950 | - |
| *RNF43* | Het | c.1504A>G (p.Ser502Gly) | 4 (D) | Missense | E3 ubiquitin ligase. Susceptibility to serrated polyposis | - | - |
| *ROBO1* | Het | c.4610G>A (p.Gly1537Glu) | 2 (D)/13 (I) | Missense | Axon guidance. Somatically mutated and expression altered in GC samples. It might function as TS gene | 168/280496 | - |
| *SDHC** | Het | c.50A>G (p.His17Arg) | 3 (D) | Missense | Subunit of succinate dehydrogenase complex. Involved in paranglioma and gastrointestinal tumors predisposition | - | - |
| *SIRT3* | Het | c.1156C>G (p.Leu386Val) | 13 (I) | Missense | ROS elimination, apoptosis inhibition and cancer formation prevention. May act as a TS in gastric cancer | - | - |
| *TLR1* | Het | c.2090T>C (p.Leu697Ser) | 10 (D) | Missense | Inflammatory response to *H. pylori* recognizing PAMPS | 4/282850 | - |
| *TLR2* | Het | c.2032G>C (p.Asp678His) | 20 (I) | Missense | Recognition of PAMPs in response to bacterial lipoproteins | - | - |
|  | Hom | c.1232C>T (p.Thr411Ile) | 4 (D) | Missense |  | 1189/281072 | - |
| *TLR5* | Het | c.1175G>A (p.Arg392Gln) | 1 (D) | Missense | Inflammatory response to *H. pylori* recognizing bacterial flagellin | 69/282546 | - |
|  | Het | c.2254A>G (p.Arg752Gly) | 5 (D) | Missense |  | 255/282772 | Benign |
| *TLR10* | Het | c.734C>A (p.Ser245*) | 3 (D) | Stop gained | Fundamental role in pathogen recognition and activation of innate immunity triggered by *H. pylori* | 114/272948 | - |
| *WWOX* | Het | c.928C>T (p.Arg310Cys) | 1 (D) | Missense | TS affected in multiple cancers. Loss of function is associated with DNA repair abnormalities | 80/280976 | Uncertain significance |
|  |  | c.1171G>A (p.Glu391Lys) | 1 (D) | Missense |  | 14/249522 | Uncertain significance |
|  |  | c.76A>C (p.Thr26Pro) | 5 (D) | Missense |  | - | - |
| *UNG* | Hom | c.466T>C (p.Tyr156His) | 8 (D) | Missense | Mutagenesis prevention | - | - |

Het, Heterozygous; Hom, Homozygous; D, diffuse histotype; I, intestinal histotype; TS, tumor suppressor; GC, gastric cancer CRC, colorectal cancer; MSI-H, microsatellite instability high; ROS, reactive oxygen species; PAMPS, Pathogen-associated molecular patterns.
