# Supplementary material for: Identification of New Genes Involved in Germline Predisposition to Early-Onset Gastric Cancer
Source: Int J Mol Sci. 2021 Jan 28;22(3):1310. doi: 10.3390/ijms22031310 (PMC7866206; doi:10.3390/ijms22031310)
Supplement: Supplementary file 1 [file ijms-22-01310-s001.zip › supp/Table S2-checked_27012021.docx]

**Table 2.** Key terms used to prioritize genetic variants with functional annotations and/or bibliography related to cancer. Functional annotations listed corresponded to those selected from Gene Ontology, KEGG and REACTOME, whereas the Bibliography category referred to terms from NCBI gene summary and Gene Reference Into Function (GeneRIF). Noteworthy, other annotations containing listed key terms were also selected (e.g. “breast cancer” since it contains “cancer”).

| **Gene ontology biological process terms** | | | | | | | | | | | |  |  |
| --- | --- | --- | --- | --- | --- | --- | --- | --- | --- | --- | --- | --- | --- |
| **Apoptosis and cell death** | | | | | | | | | | | |  |  |
| *Apoptotic process*  Execution phase of apoptosis  Apoptotic signaling pathway  Mitochondrial outer membrane permeabilization  Release of cytochrome c from mitochondria  Apoptosome assembly | | | | | *Cell death*  Programmed cell death  Autophagic cell death  Macroautophagy  Microautophagy  Regulation of autophagy  Cytolysis  Necrotic cell death | | | | | | |  |  |
| **Cell adhesion** | | | | | | | | | | | |  |  |
| Cell-cell adhesion  Homophilic cell adhesion  Cell-cell junction assembly | | | | Cell-substrate junction assembly  Regulation of cell junction assembly  Adherens junction assembly  Cell adhesion mediated by integrin | | Cell-substrate adhesion  Regulation of cell adhesión  Tight junction assembly | | | | | |  |  |
| **Cell cycle** | | | | | | | | | | | |  |  |
| Cell cycle  Cell cycle phase  Cell cycle phase transition  Mitotic cell cycle  Abortive mitotic cell cycle  Mitotic spindle organization  Mitotic nuclear envelope disassembly  Mitotic nuclear envelope reassembly | | | | Establishment of mitotic spindle localization  Mitosis  Mitotic prophase  Mitotic metaphase  Mitotic anaphase  Mitotic telophase  Mitotic prometaphase  Mitotic sister chromatid segregation | | Spindle assembly involved in mitosis  Mitotic spindle assembly checkpoint  Mitotic centrosome separation  Cell cycle arrest  Cell cycle checkpoint  DNA integrity checkpoint  Spindle assembly checkpoint | | | | | |  |  |
| **Cell differentiation** | | | | | | | | | | | |  |  |
| Cell differentiation  Epithelial cell differentiation  G1 to G0 transition involved in cell differentiation  Mesodermal cell differentiation | | | | Stem cell differentiation  Cell development  Cell fate commitment  Negative regulation of cell differentiation | | Positive regulation of cell differentiation  Regulation of epithelial cell differentiation  Regulation of epitelial to mesenchymal transition | | | | | |  |  |
| **Cell growth** | | | | | | | | | | | |  |  |
| Cell growth  Developmental cell growth  Regulation of cell proliferation | | | | | Regulation of epithelial cell proliferation  Cell motility | | | | | | | |  |
| **Cellular response to DNA damage stimulus** | | | | | | | | | | | | |  |
| DNA repair  Single strand break repair  Mismatch repair  Postreplication repair  Double-strand break repair  DNA replication proofreading  Non-recombinational repair  Base-excision repair | Nucleotide-excision repair  UV-damage excision repair  Interstrand cross-link repair  DNA damage checkpoint  DNA damage response, detection of DNA damage  DNA damage induced protein phosphorylation | | | | | | | | | | Replication fork processing  Telomere maintenance in response to DNA damage  Intrinsic apoptotic signaling pathway in response to DNA damage  Signal transduction in response to DNA damage | |  |
| **Chromatin** | | | | | | | | | | | | |  |
| Chromatin organization  Helicase activity  Maintenance of DNA methylation  Chromosome organization  Kinetochore organization  Chromosome condensation  Chromosome decondensation | Chromosome breakage  Telomere organization  Maintenance of DNA repeat elements  Sister chromatid cohesion  Regulation of cohesin localization to chromatin  Centromere complex assembly  Regulation of chromosome organization | | | | | | | | Maintenance of chromatin silencing  Telomere maintenance  Maintenance of fidelity involved in DNA-dependent DNA replication  DNA protection  Chromatin remodeling | | | |  |
| **Pathways** | | | | | | | | | | | | |  |
| Wnt receptor signaling pathway  BMP signaling pathway  SMAD protein signal transduction  SMAD protein complex assembly  Negative regulation of MAPKKK cascade  Negative regulation of MAP kinase activity  Regulation of JUN kinase activity  Negative regulation of JNK cascade  Insulin receptor signaling pathway  Cellular response to TGF-beta stimulus  Notch receptor processing, ligand-dependent | Cellular response to TGF-beta stimulus  GTPase activity  Transforming growth factor beta receptor signaling pathway  Response to transforming growth factor beta stimulus  Epidermal growth factor receptor signaling pathway  Notch signaling pathway  Positive regulation of transcription of Notch receptor target  Protein serine/threonine kinase activity  Protein kinase activity  Negative regulation of protein kinase B signaling cascade  Anterior/posterior pattern formation | | | | | | | Netrin-activated signaling pathway  Regulation of TOR signaling cascade  Target of rapamycin signaling pathway  Regulation of phosphatidylinositol 3-kinase cascade  NF-kappab import into nucleus  Regulation of NIK/NF-kappab cascade  Regulation of p52-dependent NF-kappab signaling | | | | |  |
| **KEGG pathway terms** | | | | | | | | | | | | |  |
| Base excision repair  Nucleotide excision repair  Mismatch repair  Homologous recombination  Non-homologous end-joining  Cell Cycle Checkpoint  Metabolism of xenobiotics by cytochrome P450  Benzoate degradation  Aminobenzoate degradation  Fluorobenzoate degradation  Chloroalkane and chloroalkene degradation  Chlorocyclohexane and chlorobenzene degradation | Chlorocyclohexane and chlorobenzene degradation  Toluene degradation  Xylene degradation  Nitrotoluene degradation  Ethylbenzene degradation  Styrene degradation  Atrazine degradation  Caprolactam degradation  DDT degradation  Bisphenol degradation  Dioxin degradation  Naphthalene degradation  Polycyclic aromatic hydrocarbon degradation  Furfural degradation  Steroid degradation | | | | | | | Metabolism of xenobiotics by cytochrome P450  Focal adhesion  Adherens junction  Tight junction  Gap junction  Purine metabolism  Pyrimidine metabolism  Wnt signalling pathway  Notch signalling pathway  TGF-beta signalling pathway  Insulin signalling pathway  PI3K-Akt signalling pathway  Mucin type O-Glycan biosynthesis  Hedgehog signalling pathway | | | | |  |
|  | **Reactome terms** | | | | | | |  | | | | |  |
| DNA Repair  Base Excision Repair  Cleavage of the damaged purine  Depurination  Removal of DNA patch containing abasic residue  Resolution of AP sites via the single-nucleotide replacement pathway  Resolution of Abasic Sites (AP sites), organism-specific biosystem  Gap-filling DNA repair synthesis and ligation in GG-NER  Gap-filling DNA repair synthesis and ligation in TC-NER |  | Transcription-coupled NER (TC-NER)  Repair synthesis for gap-filling by DNA polymerase in TC-NER Apoptosis  Apoptotic execution phase  Apoptotic cleavage of cellular proteins  Senescence and Autophagy  Cell Cycle, Mitotic  Mitotic Anaphase  M Phase  G1/S Transition  DNA Replication  G2/M Transition  Integrin cell surface interactions  Adherens junctions interactions  Cell-Cell communication  Telomere Maintenance | | | | |  | | | Extension of Telomeres  Chromosome Maintenance  Signaling by BMP  Signaling by TGF beta  Signaling by Wnt  Beta-catenin phosphorylation cascade  Degradation of beta-catenin by the destruction complex  O-linked glycosylation of mucins  PKB-mediated events  TGF-beta receptor signaling  Constitutive PI3K/AKT Signaling in Cancer  Chromosome Maintenance  O-linked glycosylation of mucins | | | |
| **Bibliography terms** | | | | | | | | | | | | | |
| Adenocarcinoma  Adenoma  Breast cancer  Cancer  Carcinoma  Chromosomal instability  Colon cancer  Colorectal cancer  Cowden | | | Diffuse subtype  Epstein-barr virus  Gastric adenocarcinoma and proximal polyposis of the stomach  Gastric cancer  Gastroesophageal junction  Gastrointestinal tumor  Helicobacter pylori  Intestinal subtype | | | | | Microsatellite instability  Oncogene  Polyposis  Polyps  Rectal cancer  Sarcoma  Stomach  Tumor  Tumor suppressor | | | | |  |
