# Supplementary material for: Identification of New Genes Involved in Germline Predisposition to Early-Onset Gastric Cancer
Source: Int J Mol Sci. 2021 Jan 28;22(3):1310. doi: 10.3390/ijms22031310 (PMC7866206; doi:10.3390/ijms22031310)
Supplement: Supplementary file 1 [file ijms-22-01310-s001.zip › supp/Table S3-checked_27012021.docx]

**Table S3.** Primer sequence for Sanger sequencing validation.

| **Gene** | **Variant** | **Forward primer sequence 5’ -3’** | **Reverse primer sequence 5’ -3’** | **PCR fragment size** |
| --- | --- | --- | --- | --- |
| *ADAMTS9* | c.4259T>C | AATTTGTCATTGGCCTTTCA | TGTGTGTTACACTGCTCACG | 188 |
| *APC* | c.7262C>T | GAAGTGAGTCTGCCTCCAAA | GCTTGGAGCTTCTTTGATGA | 170 |
|  | c.6958C>T | TCAGAATTAAGCCCTGTTGC | GGGATGATGTCCTTGGAAGT | 214 |
| *ARID1B* | c.3760A>G | CACGACCCATTCTCAGATGT | CTTTTCTCATTCCCCCAAAG | 154 |
| *ARID4A* | c.449T>G | GACACTTGACCAGCTTCCAT | CCAAATGAATTTAGCCATCC | 184 |
| *ATM* | c.68G>A | CAGACAGTGATGTGTGTTCTGA | CAGGCGCTTAAATTTCTCAA | 211 |
| *ATP4A* | c.2002C>T | CTGATCTGAAGGGGTCAGTG | TCGGTCTGTGCCAGATGT | 230 |
| *A4GNT* | c.719A>G | GGAATGCATGGAAAACTTTG | CTTGGCTCTGTATCCCACAC | 225 |
| *BAP1* | c.1268C>A | TCTGGGTACTGCTGGGTATG | TGGACAGAGGAATTGAGAGG | 209 |
| *BCL6B* | c.173C>T | GAGTTCACTCGCCACTCCT | AAAGACTTGGCTTCGGATTC | 221 |
| *COL7A1* | c.7334C>T | AGGGTTCCCAAGTCACTCTC | CACGAGGACAGGAAATCAAA | 170 |
| *CTNND1* | c.28_29delinsCT | TCTCTCCCTCCTTCTCCTTC | CGGGTCAGCTTCTCAAACT | 160 |
| *DACT2* | c.1784C>A | CTACCCCATGCCTGTCCT | GGGCCCCAGGTTAGACTC | 196 |
| *EPHB2* | c.847G>C | TTTCCTGGTGACTCTCCTTG | GGGCCCTAGGACTTACTTGT | 205 |
| *ERCC2* | c.1775G>A | TGGAGAAGTACCAGGAGGTG | CCAGTCCACTCACCAAAGTC | 189 |
| *EXT1* | c.770C>T | CGACCCAACTTTGATGTTTC | CATTCCTGGTGTCTGATCCT | 166 |
| *EXT2* | c.965G>A | TTTTGTGTCAAGATGCCTCA | AGGAGTCTGCAATGACAACC | 230 |
| *FANCA* | c.98C>G | GGGACATATGGCTCAACTCA | CAGGATGCTTCCATCTGTTC | 155 |
| *FAT1* | c.10195G>A | CAAGGAAGCTCGTTCACAAT | TGTTTACATGTTGCCGTCAG | 205 |
| *FAT2* | c.3311A>G | CTACAAGGGGGTGGGTATG | GAACATGACCTCCTCACAGC | 166 |
| *FAT4* | c.2186C>A | CTGCCACTGACCCAGACTT | GTGGGTTGTCTTGAGTGTCC | 237 |
| *GATA2* | c.445G>A | GTGAGCCCCTTCTCCAAG | GTGCTAGGGTCAGGAGACAC | 209 |
| *GPC3* | c.167C>T | CGCTCCTTCTTCCAGAGACT | TCTCCCTCCCTCAGTAGACC | 233 |
| *GPX7* | c.137C>T | CAGGAGCAGGACTTCTACGA | AGGTGCCGGTAGCGAAGT | 356 |
| *HBP1* | c.1167A>C | CACACCTATGGATTCTTCTGC | GCTGTGGTTTTTGACAGCTT | 190 |
| *IL12A* | c.556C>A | ATGGGGGAAATGTTTTTAGC | CCTGCATCAGCTCATCAATA | 206 |
| *IQGAP2* | c.4582G>A | ATGTTCCTTTCTTCACAGGTTT | AGAGAGAGAGCCAGGGTAGC | 204 |
| *ITIH5* | c.1400C>T | GAGAAACTGTCGCTGGAGAA | GTTGTCAGCAGATGCAAGG | 192 |
| *KAT5* | c.478G>C | AAAGGAAGACCCTGGACCTA | GTGGGACCTGAGAAGAAAGC | 231 |
| *LARP7* | c.778A>G | AGAAGAAGAAAGGCCGAATG | TTCAACTCTGTCCCGTTTTT | 185 |
| *LATS1* | c.286C>T | CCTCGACAAGTCAGAAATCC | CTCATCAAATCCAGCAGCTT | 165 |
| *LIG3* | c.86G>A | TTGGCTTTCAAGATCTTCTTTC | CGTGATCTTAGATGGCTTCC | 194 |
| *LRP1B* | c.7484G>A | AGGCTGCCATGACTTGTG | TGATGTGTGTGGAATTTGGA | 154 |
| *MAD1L1* | c.787C>T | GCGATTGTGAAGAACATGAA | CTAGGGATAAGGCCAAGAGC | 209 |
| *MUC1* | c.758C>T | ATGCCAGTAGCACTCACCAT | GCCGATACTCACCATTTCAG | 203 |
| *NEO1* | c.4090G>T | TCTTCGTCTCAAACATGCTG | GGGACAGTAATGGTGTGCTT | 217 |
| *PHF2* | c.2188A>G | AAGTATCCTCCTCCCAACTCC | AGCTCCCACTCTCCTTCTTG | 170 |
| *POLD1* | c.931C>T | GCTGTGGTCTGACGTGGT | ACCTTCATGCTTGCCATTC | 198 |
| *POLH* | c.698A>G | TACTTTGCATGCTTCCAGGT | GAAATTTAACATGCTGCCTGA | 151 |
| *PTCH1* | c.3924A>T | GGGATACCACCCTTCTAACC | CCAGAATGCCCTTCAGTAGA | 229 |
| *POT1* | c.143C>T | TGAAAGCCAAGAAAATGTCTC | TCCACTAAAGAGCAGGCAAG | 219 |
| *RAD23A* | c.434G>A | CATAAGTGGTCCCACACACC | GAAGAGGAAAGGGCAGCTC | 234 |
| *RCC1* | c.206T>C | GGGTGAGAATGTGATGGAGA | GAACATGCGTGGTTCAAAAG | 194 |
|  | c.508C>T | AGCTGCAAGAGAAGGTGGTA | TTTCATCTCCCCACCAGTAA | 208 |
| *RNF43* | c.1504A>G | CATGGCTCTTCCAGTGACTC | ACACTAGGCTGCATGTCCAC | 164 |
| *ROBO1* | c.4610G>A | TGCCAAAACTCCCTTCTATG | TTTGCTGTTCCTGTGCTTCT | 150 |
| *SDHC* | c.50A>G | TTATCTTGCAGACACGTTGG | ATGGCGTGAACCCAAGAG | 199 |
| *SIRT3* | c.1156C>G | CGTGGAAAGCCTAGTGGAG | GTAGTGCCTGGTGCAGGAT | 213 |
| *TLR1* | c.2090T>C | AACTTTGTTCCTGGCAAGAG | GAATGGAGTACTGCGGAATG | 205 |
| *TLR2* | c.2032G>C | CCAGCAGGAACATCTGCTAT | TTTGTGGCTCTTTTCAATGG | 191 |
|  | c.1232C>T | CCTGGCCCTCTCTACAAACT | CATCTTTTCTGGCCACTGAC | 161 |
| *TLR5* | c.1175G>A | ACCTTCTGGGGGAACTTTAC | GATATCGGGTATGCTTGGAA | 188 |
|  | c.2254A>G | CTTTGTCCCAGGAGAAAACC | CACCATGATGAGAGCACTGT | 172 |
| *TLR10* | c.734C>A | ATGGCAAAAGCCAATTTGTA | CGGATCTGAAAGTGTTCCAC | 163 |
| *WWOX* | c.928C>T | GCTGGCTTATAACAGGTCCA | GGTTTCTCTAGGACCCAAGG | 237 |
|  | c.1171G>A | TTCTTGGATTTCCAGCAACA | TTCTTGGATCAGCCTCTCG | 180 |
|  | c.76A>C | GCTGGACGACACGGACAG | CCTTTTCCCTGAAGCTCCTT | 227 |
| *UNG* | c.466T>C | GGGTCTGTGCTGCTTACATT | AGGAATCATGTCTGGACTGC | 199 |
