# Supplementary figures and images for: Identification of New Genes Involved in Germline Predisposition to Early-Onset Gastric Cancer
Source: Int J Mol Sci. 2021 Jan 28;22(3):1310. doi: 10.3390/ijms22031310 (PMC7866206; doi:10.3390/ijms22031310)

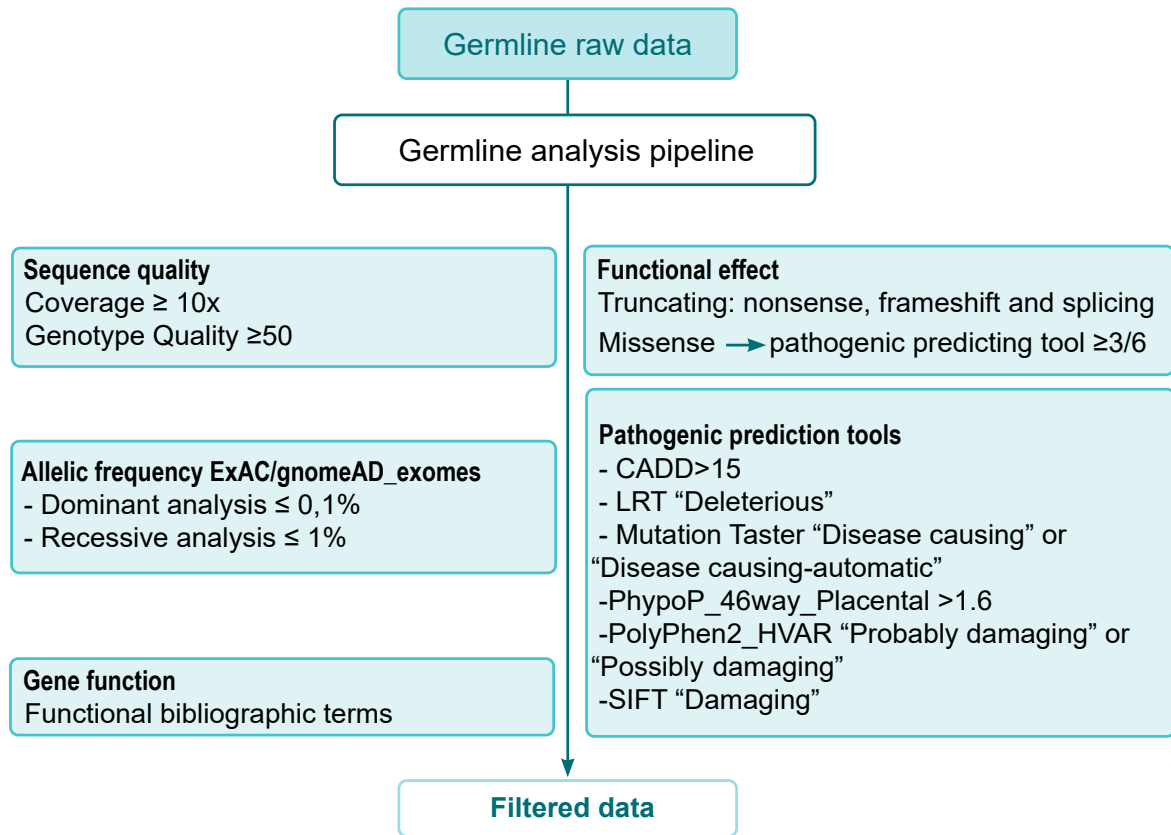

Supplement: Supplementary file 1 [file ijms-22-01310-s001.zip › supp/Supplementary Figure S1.pdf]
